# Preliminary Study on Mechanical Aspects of 3D-Printed PLA-TPU Composites

**DOI:** 10.3390/ma15072364

**Published:** 2022-03-23

**Authors:** Alicja Żur, Paweł Żur, Piotr Michalski, Andrzej Baier

**Affiliations:** Department of Engineering Processes Automation and Integrated Manufacturing Systems, Faculty of Mechanical Engineering, Silesian University of Technology, 44-100 Gliwice, Poland; pawel.zur@polsl.pl (P.Ż.); piotr.michalski@polsl.pl (P.M.); andrzej.baier@polsl.pl (A.B.)

**Keywords:** 3D printing, composite, FDM, FEM analysis, motion simulation, vibrodiagnostics

## Abstract

Additive technologies using Fused Deposition Modeling (FDM) technology are currently a promising tool for the production of polymeric multicomposites. This paper presents the results of a static 3-point bending test carried out on 3D printed samples of the PLA-TPU composite. The article also discusses initial vibrodiagnostic research and Finite Element Method (FEM) analysis of the 3D-printed composite bushings. The data obtained from FEM analysis served as input data for motion simulation analysis, where the influence of the stiffness of the suspension on the trajectory has been verified.

## 1. Introduction

In recent years, the role of composite materials as engineering materials has increased significantly. Currently, composites are widely used in construction and transport, especially in the automotive, aviation, marine, and sports industries. For example, composite materials used in aircraft until the 1980s accounted for no more than 5% of the total weight of the aircraft. Currently, more than 50% of the total weight of the aircraft is made up of composite materials [1].

Currently, new technologies for producing composites in conjunction with new materials allow us to replace traditional materials with light structures of similar strength. These structures allow one to reduce the weight and material needed to fabricate the element. Additive Manufacturing (AM), colloquially known as 3D printing, is among those manufacturing methods that have attracted the attention of both industry and scientists over the past few years [2].

Manufacturing a multicomponent material is a complex process. Fused Deposition Modeling (FDM) was used in this study to reduce the effort required to produce the component. As a result of the use of a multiextruder, a composite element was obtained as a single printout. In addition to performing traditional tensile bending strength tests, the stiffness value of the composite bushing suitable for use in a Greenpower class electric car was determined. [3]

**Figure 1:**
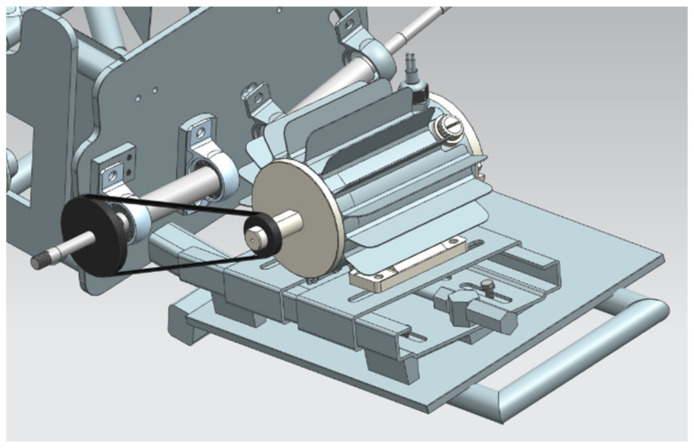
Geometry of the suspension and drive shaft of the electric vehicle of the Silesian Greenpower team.

The suspension used so far in the Silesian Greenpower team’s race car was characterized by very high stiffness. Until now, metal bushings have been used to support the shafts, both the drive shaft and the passive shaft on the other side of the car. It is worth mentioning that this shaft does not rotate; at its end is mounted a wheel with a bearing, which allows the wheel to move. The suspension geometry including the driveshaft is shown in Figure 1. The illustration shows the car’s drivetrain, which is located at the rear of the vehicle. The motor, shielded by a radiator, is mounted behind the driver’s cab. The vehicle has one driven wheel that is connected to the engine by a timing belt. The picture shows the drivetrain without the wheel so as not to obstruct the view of the drivetrain. The use of a completely rigid suspension was dictated by the desire to minimize the loss of mechanical energy. However, the lack of any elastic or damping elements in the suspension transmits any vibrations arising from uneven surfaces, for example, to the body of the car and the frame of the car, and thus affects the driving conditions of the driver during the race [4,5,6,7]. 

The main objective of the conducted research was to investigate the purposefulness of using a PLA-TPU composite bushing. It is intended that this bushing will replace the steel bushing previously used in the suspension. The purpose of this study was initially to verify the properties of an elastic composite suspension bushing. The study initially checked the effect of changing the metal bushing to a flexible composite bushing on the handling properties of the car at one of the selected corners of the track.

Metal-rubber bushings are commonly used in suspension systems. Here, however, it is possible to design the rubber filling of the bushing so that it affects the desired trajectory of the vehicle. This allows for custom solutions to be created to match the track and the corners being taken.

## 2. Materials and Methods

In this section, the test methodology of the conducted tests is described. First, the experimental test of static bending of PLA-TPU composite specimens with different fillings was carried out. Then the methodology for performing vibrodiagnostic research and numerical analysis of the stiffness values for different bushings in the ANSYS Mechanical 19.2 Academic software were explained. Finally, the method for modeling the car ride on the track is described using the parameters of the composite bushing in the Siemens NX v.11 program.

### 2.1. Experimental Static Bending Test

The first step of this study was to make 3D prints of composite samples. Samples were PLA 3D prints with a rubber core. PLA (polylactid acid) material is a commonly used thermoplastic for FDM technology prints. This is due to the wide availability and biodegradability. PLA has one of the highest thermal capacities among polyester polymer materials [2,6,7]. PLA has a variety of applications and tends to be influenced by different processing methods and storage conditions. Currently, a large amount of research has been devoted to developing various polylactide changes to make the material suitable for a wider range of products. In many cases, PLA modification is made by copolymerization, surface treatment, or stereocomplexation. The mixture drastically changes the properties and degradation patterns of the intrinsic polymer and thus affects its adaptability to different end-use applications [8,9,10,11,12,13,14]. Since these fibers are of a hollow and lignocellulosic nature, they have very good thermal and acoustic insulation properties. Because of their low density, low cost, and high specific modules, they attract attention from the industry [15]. The PLA filament used in this study was Spectrum’s PLA.

The material that was the flexible core of the samples was also a thermoplastic polymer, Extrudr TPU Medium. TPU is a thermoplastic polyurethane whose basic feature is high flexibility. One of the characteristics of thermoplastic polyurethane is abrasion resistance. This material also absorbs shocks. It is resistant to oils, solvents, and greases [4]. Thermal polyurethane elastomers (TPUs) are a thermoplastic elastomer (TPE), which combines thermal rubber mechanical properties with thermal polymer processing capabilities. Because there are usually no chemical networks in rubber, they can melt and process repeatedly. The TPU was the first homogeneous and thermoplasticized elastomer to be processed [16].

TPU is a flexible elastomer that offers flexibility to the composite. Its glass transition temperature (Tg) is −50 °C (based on DSC test), weight average molecular weight (Mw) is 108.5 kDa, and polydispersity index (PDI) is 2.26 (based on GPC test). PLA was selected to improve the rigidity of the composite. Its Tg is 60 °C (based on DSC test), Mw is 127.1 kDa, and PDI is 1.6 (based on GPC test). Both TPU and PLA have similar melt processing windows, allowing them to be compounded properly [17].

The samples were printed on a Prusa MK3 printer with a multiextruder feature. Eight series were printed, each series consisting of six samples. The series differed in pattern and filling density (Table 1), which have been selected based on previous research [6]. The shape of the samples (80 mm × 4 mm × 10 mm rectangle) was prepared according to the standard PN-EN ISO 178: 2011 Plastics—determination of flexural properties. As this is a preliminary study, the thickness of the outer layer was initially assumed to be 0.8 mm for each series. The print parameters (nozzle temperature and bed temperature) were set according to the recommendations of the filament manufacturer. An important factor in the process of preparing the prints was the appropriate selection of the ramming parameter. This parameter is responsible for the shape of the filament tip when changing the material during printing. This is an important factor when printing with two materials of different flexibility.

The printed samples were subjected to a three-point static bending in accordance with the PN-EN ISO 178: 2011 standard. The machine used for the static bending test was a Zwick Roell Z020. The three-point bending test stand consisted of two supports with a radius of 5 mm and a spacing of 64 mm, and an arm to which the load was applied, also with a radius of 5 mm.

### 2.2. Finite Element Method Analysis

**Figure 2:**
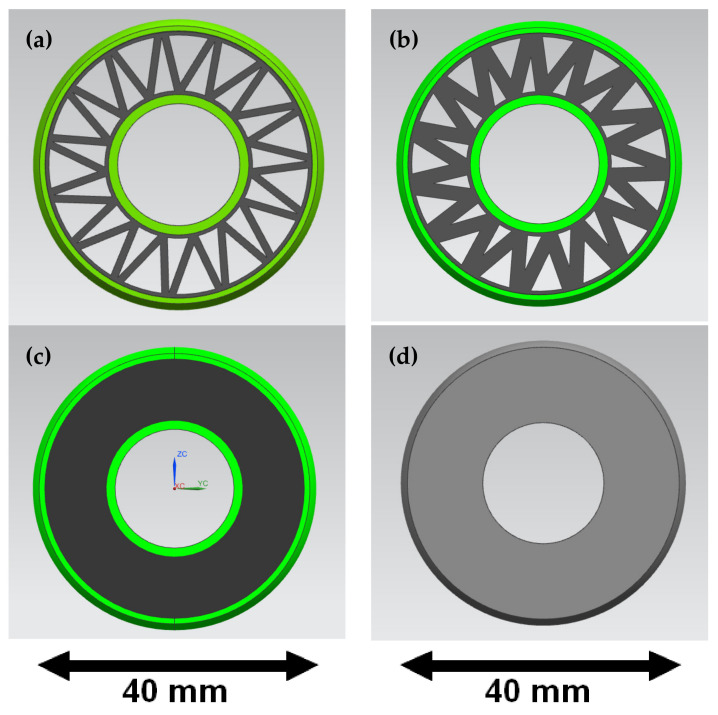
Geometries of the bushings—PLA-TPU with 1 mm V-shaped filling (**a**), PLA-TPU with 2 mm V-shaped filling (**b**), PLA-TPU with full TPU filling (**c**), steel bushing (**d**).

The second phase of this research was the Finite Element Method analysis of the 3D-printed bushings [8,9,10,11]. FEM analysis was performed in ANSYS 19.2. Academic software. In total, four models were compared. Three models of the FDM printed PLA-TPU bushing and one model of a simple steel bushing (for reference only), which is still applied today in the Silesian Greenpower vehicle. The CAD models of the FDM printed bushing represented two different materials: the 1mm outer layers of PLA and a TPU rubber filling. Different types of TPU fillings were chosen: a full filling and a V-shaped pattern with two different thicknesses. The V-shaped filling pattern was specifically designed to reduce the needed material and printing time while providing optimal mechanical properties. Two variants of the V-shape infill pattern were inspected, 1 mm thick and 2 mm thick. The geometries of the PLA-TPU bushings are presented in Figure 2.

Initially, the 3D printed bushing was subjected to compressing force on the hydraulic press equipped with a high precision load cell. Then the deflection could be measured. Having the concept of the test stand, the same conditions were modeled in ANSYS Mechanical v19.2 Academic software by applying corresponding boundary conditions. The lower support of the press was represented by a fixed constraint, and the upper arm of the press was represented by applying a perpendicular force. The bushing was deflected in the direction perpendicular to the axial direction of the element. All elements were subjected to the same force value—200 N, so the values obtained of the stiffness coefficient could be compared.

Knowing the force acting on the element and the deflection, the stiffness coefficient *k* could be calculated using Equation (1),

(1) $$k=\frac{F}{\delta }$$

where:

*F* [N]—force acting on the body,

*δ* [mm]—the displacement produced by the force in the same direction.

Then, the values of the stiffness coefficient obtained for different types of bushings were used in the motion simulation analysis to verify the trajectory of the car.

### 2.3. Vibrodiagnostic Research

**Figure 3:**
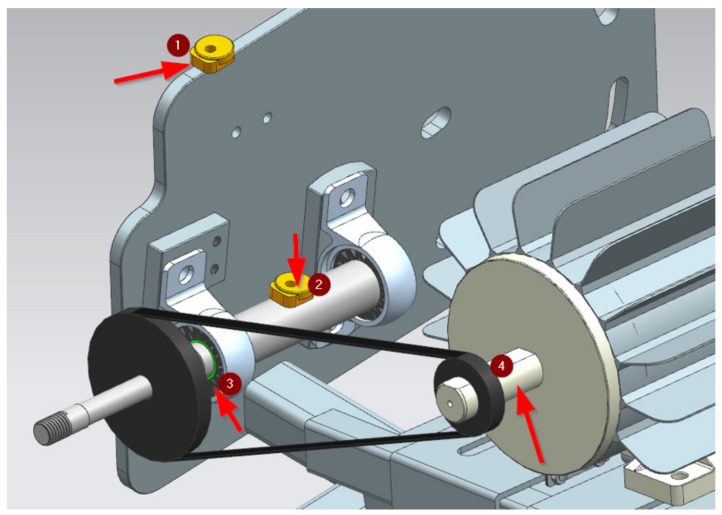
Test stand and equipment: 1—VSA003 reference sensor, 2—VSA003 sensor, 3—PLA-TPU bushing, 4—motor shaft.

The designed and developed rubber mounts were subjected to vibrodiagnostic tests in the environment of the target workplace, in the racing car. After the shaft of the wheel was mounted on the car frame of the mechanical system, the measuring equipment was put on. During the tests, a VSE100 vibrodiagnostic unit from IFM electronic was used. Diagnostic electronics have two analog inputs and four dynamic inputs. These inputs can be used for process value monitoring, vibration monitoring, vibration diagnostics, or analysis of other dynamic signals. The test stand with the used equipment is presented in Figure 3.

In the described tests, one analog input and two dynamic inputs were used. The analog input was used to continuously determine the rotational speed of the car wheel mounted on the shaft. In the spectrum of signals, three characteristic harmonics (frequencies) were expected from the SKF6002 bearing used in this wheel, which are: inner race, outer race, and rolling elements of the bearing. Additionally, two VSA003 series piezoelectric vibration sensors were mounted on the wheel shaft and on the car’s support frame. These signals are connected to the dynamic inputs of the evaluation unit and are continuously detected and permanently monitored according to the set tasks (parameters). The monitoring tasks of the process values and objects were defined on the PC by means of dedicated software VES004 and then transferred to the diagnostic electronics as parameter set via Ethernet interface. The characteristic values in the frequency range were monitored in sequence (typical multiplex mode), while the time range was monitored simultaneously.

### 2.4. Motion Simulation Analysis

The last phase of this research was the initial verification of the application of the composite bushing. To this end, a study was conducted on the advisability of using an elastic bushing in the suspension of the Silesian Greenpower car.

The comfort of the ride and the road behavior of a vehicle are affected by the proper alignment of the suspension components. The ride comfort and the road behavior of a vehicle are influenced by the type of wheel suspension and the stiffness of the frame. Vibrations are an important contributor to driving comfort. The human body is sensitive to vibrations in the frequency range of 1–80 Hz. The directions of vibrations may vary, and the constructor should, as far as possible, eliminate them one by one. Vertical vibration due to lateral irregularities in the frequency range of 1 to 4 Hz is influenced by body weight, suspension stiffness, and body frequency. The softer the suspension, the lower the natural frequency, and the more comfortable the ride. Soft suspensions have side effects, such as longitudinal sway during braking and significant body roll at corners. The stiffness of the suspension is related to the stability and steerability of the vehicle, that is, safety. Firm suspension is a proven means of improving driving dynamics. Due to it, it is easier to keep the car on the chosen trajectory. In the case of the Silesian Greenpower cars in the F24+ class running with a power deficit in the 1 h race (240 W motor and 2 batteries 36 Ah), a very important aspect is the most rigid suspension causing the least energy loss of the moving vehicle [18,19,20,21,22,23,24,25,26,27,28,29,30,31].

The research carried out and described in this paper is a preliminary study indicating the possibility of designing and manufacturing a PLA-TPU flexible bushing capable of being mounted in a vehicle suspension and having a significant impact on vehicle trajectory changes with the least possible energy loss.

**Figure 4:**
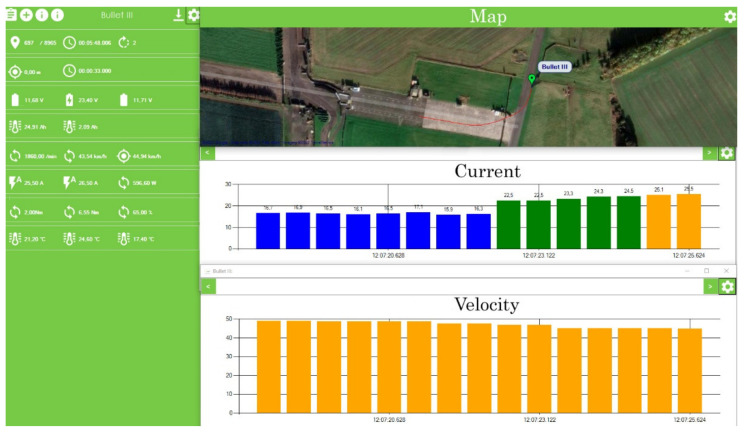
View of the parameters of the actual car run presented in the Silesian Greenpower telemetry system [12].

A preliminary simulation study was conducted on one of the tracks where the passing parameters of the Silesian Greenpower cars were recorded. One of the corners of the East Fortune track was chosen (Figure 4).

**Figure 5:**
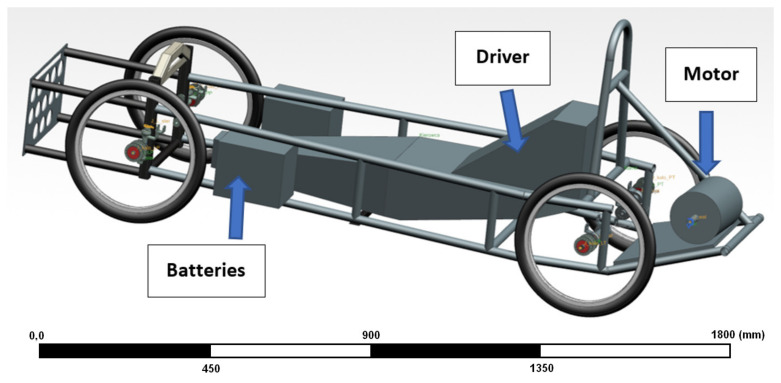
3D model of the electric car used in the simulation.

The research was carried out in a virtual environment, in which the car and the track were modeled. Modeling was carried out in the Siemens NX program in the Motion Simulation module. The aim of the preliminary tests was to compare the trajectory of the car with the flexible bushing mounted in the suspension with the trajectory of the car without the bushing and rigid suspension. In the first phase of modeling, the parameters of the model without the flexible bushing were tuned to best match the parameters of the real vehicle. These parameters were read from the Silesian Greenpower telemetry system. After model tuning, preliminary comparative tests of the car trajectory with and without the flexible bushing were carried out. The geometry of the model used in the simulation is presented in Figure 5. The model represents the overall vehicle with block models, which represent the mass distribution of heaviest elements—batteries, driver, motor. Each of the elements has been marked in the Figure below.

The results of preliminary tests showed a significant difference in the trajectories between the car with and without 3D printed bushings. The promising results of the preliminary tests were a basis for conducting a more detailed study of the effect of the bushing placement on the car trajectory. The parameters obtained from the experimental studies concerning the elasticity of the printed bushing were applied in the subsequent studies. Additionally, the steering parameters were chosen in such a way that the result of each test would not cause the car to fall out of the track. 

Four variants of bush mounting were determined. These variants are presented in Table 2. For variants 1 all bushings applied in the suspension are steel bushings. In variants 2 and 3, where PLA-TPU bushings were applied only on one side, the other side contains steel bushings. For the purpose of the next simulations, a car was accelerated to a speed of 65 km/h. The car reached this speed before entering the turn. For each of the variants, one steering angle was set [12,13]. 

In addition, the model car was equipped with displacement sensors.

## 3. Results

This section successively describes the results from the experimental static bending test, then from the modal and random vibration analysis, and from the experimental vibroacoustic analysis. The last section describes the results from a simulation run of a bolide on a track using a flexible composite bushing.

### 3.1. Experimental Static Bending Test

As a result of static bending, the following characteristics were determined: Young’s modulus, maximum force, deflection at maximum force, and strain (Table 3). For each mean value, the standard and percentile relative deviation were calculated.

The values for non-full fill patterns ranged from 1.23 to 1.45 GPa. It can be seen that Young’s modulus for the non-full fill pattern represents a higher value for the honeycomb pattern than for the grid pattern of the same density. The least noticed difference in Young’s modulus value for the same density patterns was for 25% infill—the difference between honeycomb and grid was only 1.5%. Higher differences—6.2% and 7.3%, respectively—were obtained for 50% fill and 10% fill. The value of Young’s modulus is significantly higher for the 100% infill series—1.93 GPa for the perpendicular pattern and 2.11 GPa for the parallel pattern. The value for parallel infill is 37% and for the perpendicular pattern 25% higher than for the 50% infill honeycomb pattern.

The maximum force observed in this study occurred for 100% fill samples—75.25 N for the parallel pattern. The parallel pattern could withstand a maximum force 6.9% higher than the perpendicular pattern. The difference in value for full and non-full infills is significant—53% compared to 50% honeycomb infill, which represented 49.22 N.

The deflection values varied in the range of 2 mm between 3.83 mm for the 10% grid pattern to 5.84 mm for the 100% perpendicular pattern. Again, a few percentile higher deflection value was noted for the honeycomb infill pattern than for a grid pattern of the same density. The difference between both full-fill patterns was 7.9%.

The von Mises stress values varied between 0.97 MPa for the 25% grid pattern and 1.87 MPa for the 100% parallel pattern. The stress values for the 25% and 10% grid patterns were almost identical; the difference between them was 1%.

On the basis of the above data, another characteristic was calculated: stiffness *k*, which is an important factor, considering the samples, to represent a suspension element. The stiffness values were calculated for the force value of 20 N. Stiffness values are presented in Table 4.

A slight increase in stiffness was observed along with the infill density. Differences between the stiffness of the honeycomb pattern and the grid pattern of the same densities are less than 9%. A significant change was observed for the full fill. The stiffness value for the 100% parallel pattern is 40% higher than for the highest non-full pattern—50% honeycomb infill of value 15.32 N/mm. The highest stiffness of 21.44 N/mm represents the 100% parallel infill.

### 3.2. Finite Element Method Analysis

As a result of the numerical Finite Element Method analysis carried out in the ANSYS software, the values of deformation and von Mises stress for each bushing were obtained. The results acquired for each bushing type are presented in Table 5.

**Figure 6:**
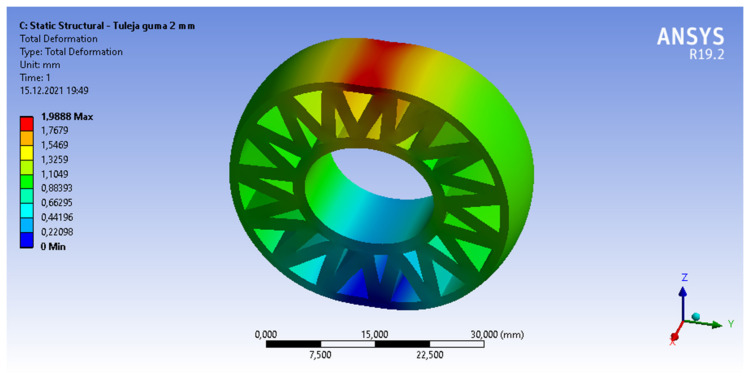
Deformation of the PLA-TPU bushing with 2 mm thick V-shaped filling at 200 N force.

It can be seen that as the thickness of the infill increases, the deformation under the applied force decreases. The deformation obtained for the model with 2 mm filling (Figure 6) is 69% lower than for the model with 1 mm filling. The difference in the stress values for these models is not as great at 32%. The deformation of the model with full infill is almost two times lower than that of the model with 2 mm infill and more than three times lower than that of the model with 1 mm infill. The stresses for the model with full TPU infill are 22% lower than for the model with 2 mm infill and 61% lower than for the model with 1 mm infill. Calculations for the steel bushing model used so far were carried out as a reference model.

Based on the deformation values obtained in the FEM analysis, the stiffness values were calculated and presented in Table 6.

An increase in stiffness was observed as the thickness of the TPU infill increased. Since all models were subjected to a force of 200 N and the stiffness was calculated from the value of this force and the deformation obtained from the FEM analysis, the percentage differences in the stiffness values are the same as the percentage differences between the deformations obtained. These results served as input to the motion simulation.

### 3.3. Vibrodiagnostic Research

The software allowed to filter the diagnostic signals, which is useful for monitoring according to ISO 10816 in the time domain, which, as the authors expected, should include the information about the changes of vibration for which the rubber binding is responsible. For these objects, the filter needs to be configured based on the rotational speed, which is, in our case, in the range of 0 to 780 RPM. During the test, we used two different filters. For rotational speed from 120 to 600 RPM, a 2Hz high-pass filter was used, and for the second range from 600 to 780 RPM the 10 Hz high-pass filter was used. Those filers were automatically switched by the diagnostic software based on the reference speed signal (optical sensor—one pulse per revolution of the racing car wheel connected to the analog input) for the diagnostic electronics. In the object, the damage frequencies are specified as the frequency factor. The actual calculation of the damage frequency was made by multiplying the frequency factor by the rotational frequency of the car wheel determined by the trigger.

**Figure 7:**
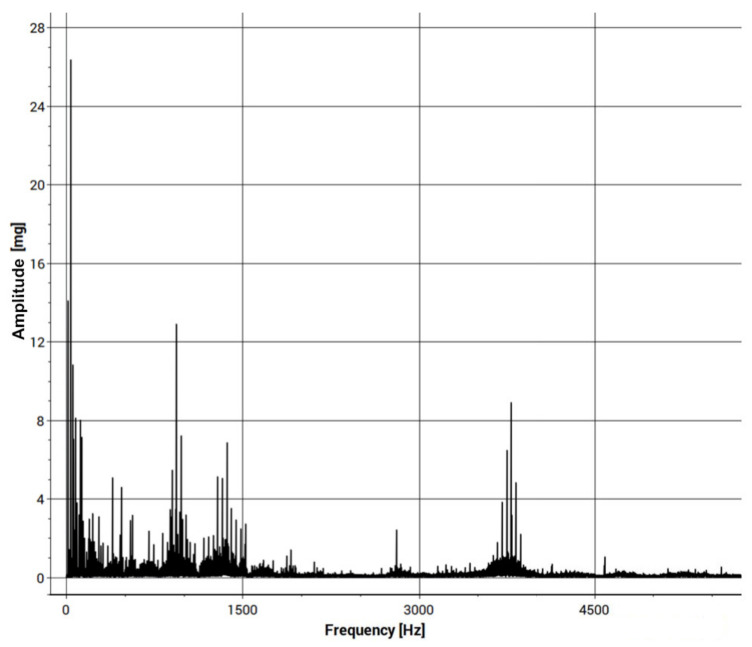
Fast Fourier Transformation of the input signal.

The results of changing the rubber bindings should be visible in the first three damage frequencies of the Fast Fourier Transformation, (soft food and loose fitting as an FFT: 1.0 fn, 2.0 fn, 3.0 fn, or misalignment in FFT: 2.0 fn). FFT is typically used for harmonic signals (e.g., in our case, unbalance of the shaft) and H-FFT (enveloped spectrum of FFT) for periodic signals (e.g., rolling element bearings of the car wheel). Taking this into consideration, two types of analyses were conducted. The results of one of them (FFT) are presented in Figure 7.

The obtained results confirm the occurrence of an increase in signal activity near the expected harmonics. Therefore, the developed research method will allow for subsequent comparative studies. Also, the results obtained confirmed three characteristic frequencies that were expected from the SKF6002 bearing.

### 3.4. Motion Simulation Analysis

**Figure 8:**
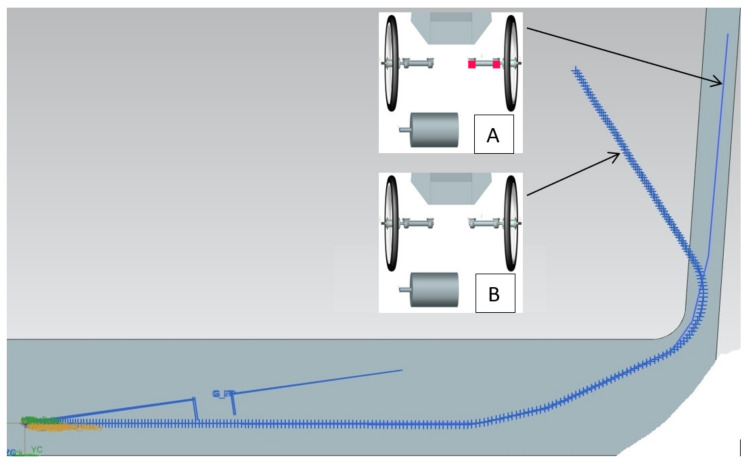
Comparison of the track of the car without (thick line) and with the elastic bushing (thin line).

A comparison of the track of the car with and without the flexible bushing is shown in Figure 8. The trajectory of the ride with the metal bushing is represented by the thick line, while that with the flexible PLA-TPU sleeve is represented by the thin line. Preliminary tests have shown that, as expected, the introduction of some additional compliance in the suspension of the Silesian Greenpower car caused a significant change in the behavior of the car in the selected corner. It is necessary to continue testing with different levels of suspension susceptibility, still studying the impact of changes first in the virtual environment and then on the track.

Significant differences in vehicle trajectory were observed. The thin line implies the racing line of the car with the elastic suspension. It can be seen that adding an elastic bushing on the outer side of the car results in a wider turning radius.

**Figure 9:**
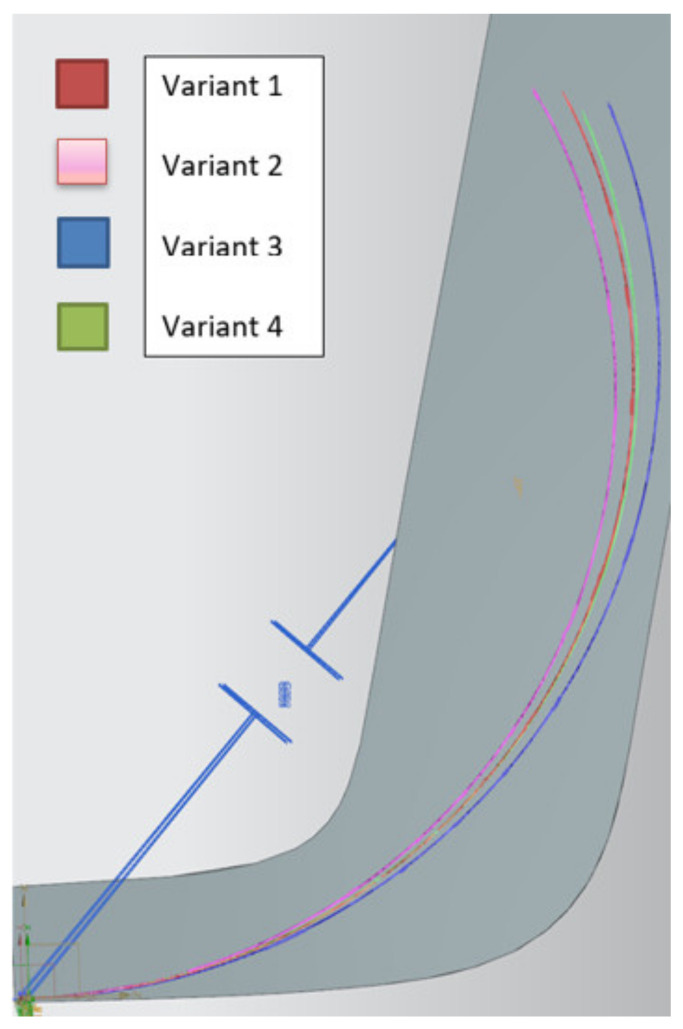
Comparison of the trajectory with different variants of PLA-TPU bushings.

The results of runs with different PLA-TPU sleeve variants are shown in Figure 9.

**Figure 10:**
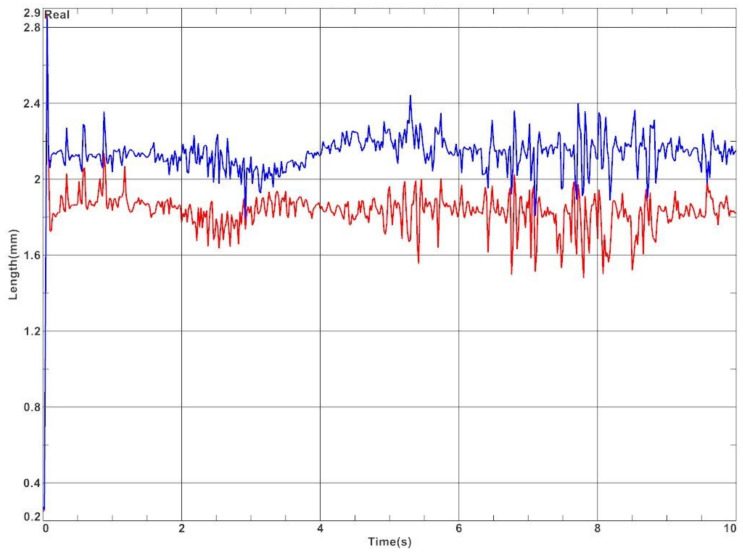
Displacement of the bushing (red—left side of the car, blue—right side of the car).

In addition, the car model was equipped with displacement sensors. Displacement values during cornering are presented in Figure 10. Displacements are shown only for variant no. 4.

**Figure 11:**
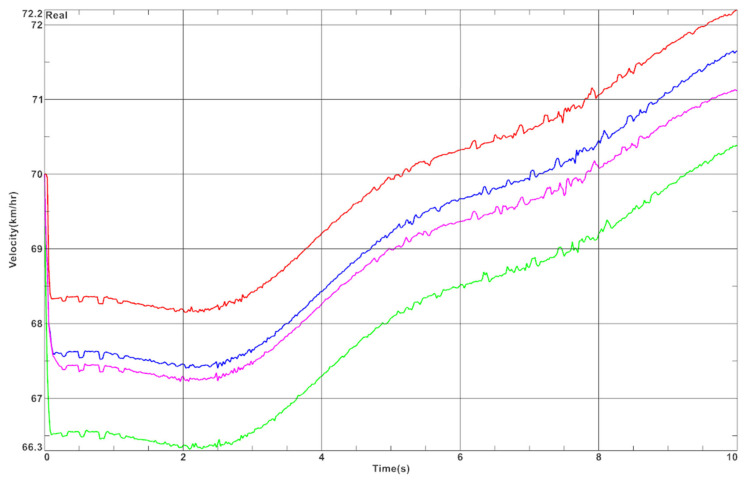
Differences in speed of the vehicle along the curve for four bushing variants (colors correspond to these presented in Figure 9).

From the graph in Figure 10, it is evident that there is more deflection on the right side, which is the outer bushing in this case. The deflection ranges from 1.8 mm to 2.4 mm. The inner bushing, on the other hand, is deflected from 1.5 to 2.1 mm. The difference between the sides is due to the centrifugal force. The differences in speed of the car along the curve for four bushing variants are presented in Figure 11 and Table 7.

On the basis of the graph in Figure 11 and data presented in Table 7, we can see that the biggest influence on the speed of the vehicle moving through the curve has the first moments of the simulation. In variant 4, a very large decrease of velocity was observed at the beginning of the simulation, while in variant 1, a small decrease of velocity was observed. This is because some energy is absorbed by the elastic bushings, which are not present in variant 1. Based on the results of the tests in Figure 2, it can be concluded that variant 3 gains speed faster than variant 2. Therefore, in the case of using elastic bushings, different variants should be considered depending on the number and type of turns that occur on the race track.

The conducted research has shown that it is possible to determine the parameters of the bushings for which the ride on the track will provide a compromise between the maximum stiffening of the car and obtaining a suspension with the assumed deflection, allowing for sporty cornering with minimal damping of vibrations affecting the comfort of driving, while not increasing losses.

## 4. Discussion

In this study, a static bending test of composite samples 3D printed using FDM technology was conducted. This allowed the determination of material properties and stiffness for components consisting of PLA with different TPU fill. The application of 3D printing technology using a multi-extruder allowed the fabrication of a composite suspension spring element. Due to the availability of this technology, it is possible to make customized components to match the requirements of a Greenpower electric vehicle.

FEM analysis was performed for four bushing models. The bushings were subjected to a force of 200 N. The analysis yielded von Mises stresses and deformation values. From these data, the stiffness factor k was calculated for each bushing. These data served as input to motion simulations, where the task was to verify the effect of the stiffness of each bushing type on the track trajectory.

The obtained results of the vibrodiagnostic research confirmed the occurrence of an increase in signal activity near the expected harmonics. Therefore, the developed research method will allow for subsequent comparative studies.

On the basis of the above results and conclusions, a preliminary motion simulation analysis was conducted to determine the effect of changing the suspension bushing from steel to PLA-TPU composite. The analysis was carried out by simulating the car driving around a selected corner of the East Fortune track. During the simulation, a significant difference was observed in vehicle trajectory through the turn. Increasing the compliance and spring rate of the suspension system allows for control of the handling characteristics of the car.

It is necessary to continue research on the use of bushings with different elastic properties, modeling the driving properties depending on the required compliance or required turning ability on the given tracks. It is also necessary to verify the influence of the susceptibility of the energy system on the potential losses of kinetic energy during driving. Additionally, the modal analysis should be performed in order to verify the application range of such elements.

## Figures and Tables

**Table 1:** Infill patterns of each printed series.

Series No.	Infill Pattern	Series No.	Infill Pattern
1.	10% Honeycomb	5.	25% Grid
2.	25% Honeycomb	6.	50% Grid
3.	50% Honeycomb	7.	100% Parallel
4.	10% Grid	8.	100% Perpendicular

**Table 2:** Variants of the possible bushing placement.

Variant 1	Variant 2	Variant 3	Variant 4
Without PLA-TPU bushings	PLA-TPU bushings on the left side (motor side)	PLA-TPU bushing on the right side	PLA-TPU bushings on both sides
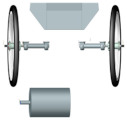	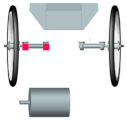	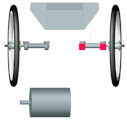	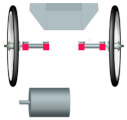

**Table 3:** Results of the static bending test (main results are highlighted in bold).

	E [GPa]			F_MAX_ [N]			d_L_ at F_MAX_ [mm]			σ [MPa]		
	${\bar{x\;}}^{a}$	$s^{\;b}$	$\nu^{\;c}$	${\bar{x\;}}^{a}$	$s^{\;b}$	$\nu {\;}^{c}$	${\bar{x}}^{\;a}$	$s^{\;b}$	$\nu^{\;c}$	${\bar{x}}^{\;a}$	$s{\;}^{b}$	$\nu^{\;c}$
Grid 10%	**1.23**	0.12	9.81	**40.42**	3.02	7.46	**3.83**	0.25	6.58	**0.98**	0.03	3.38
Honeycomb 10%	**1.32**	0.13	9.47	**41.84**	0.77	1.84	**4.08**	0.20	4.99	**1.01**	0.03	2.86
Grid 25%	**1.37**	0.13	9.21	**40.27**	2.35	5.84	**3.89**	0.62	16.03	**0.97**	0.07	7.03
Honeycomb 25%	**1.39**	0.14	10.01	**42.49**	1.69	3.99	**4.13**	0.50	12.17	**1.04**	0.04	4.24
Grid 50%	**1.45**	0.17	11.43	**45.20**	3.39	7.50	**4.63**	0.68	14.77	**1.14**	0.11	9.73
Honeycomb 50%	**1.54**	0.07	4.34	**49.22**	0.54	1.09	**4.76**	0.57	12.05	**1.21**	0.02	1.85
Parallel 100%	**2.11**	0.05	2.52	**75.25**	3.01	4.00	**5.41**	0.26	4.79	**1.87**	0.09	4.91
Perpendicular 100%	**1.93**	0.04	1.87	**70.37**	0.34	0.49	**5.84**	0.22	3.84	**1.72**	0.01	0.73

^a^ Arithmetic average. ^b^ Standard deviation. ^c^ Percentage relative deviation.

**Table 4:** Stiffness *k* values of each series at 20 N.

Infill	Stiffness *k* [N/mm]	Infill	Stiffness *k* [N/mm]
10% G	13.20	50% G	13.69
10% H	13.41	50% H	15.32
25% G	13.74	100% II	21.44
25% H	14.09	100% X	20.69

**Table 5:** Deformation and von Mises stress of each bushing at 200 N force.

Bushing Type	Deformation [mm]	Von Mises Stress [MPa]
PLA-TPU V-shape 1 mm	3.36 mm	103.96 MPa
PLA-TPU V-shape 2 mm	1.99 mm	78.75 MPa
Full TPU filling	1.09 mm	64.55 MPa
Steel	9.09 × 10^−5^ mm	25.57 MPa

**Table 6:** Stiffness *k* values of each series at 200 N.

Bushing Type	Stiffness *k* [N/mm]
PLA-TPU V-shape 1 mm	59.52
PLA-TPU V-shape 2 mm	100.50
Full TPU filling	183.49
Steel	2,200,220.02

**Table 7:** Values read from the sensors.

Variant	Initial Velocity [km/h]	Ending Velocity [km/h]	Difference [km/h]	Bushing Deflection [mm]
1	68.35	72.11	+3.76	-
2	67.45	71.48	+4.04	1.85
3	67.44	71.11	+3.67	0.4
4	66.55	70.46	+3.91	2.3|1.7

## Data Availability

Not applicable.
